# Utilizing Molecular Epidemiology and Citizen Science for the Surveillance of Lagoviruses in Australia

**DOI:** 10.3390/v15122348

**Published:** 2023-11-29

**Authors:** Nias Y. G. Peng, Robyn N. Hall, Nina Huang, Peter West, Tarnya E. Cox, Jackie E. Mahar, Hugh Mason, Susan Campbell, Tiffany O’Connor, Andrew J. Read, Kandarp K. Patel, Patrick L. Taggart, Ina L. Smith, Tanja Strive, Maria Jenckel

**Affiliations:** 1 Commonwealth Scientific and Industrial Research Organisation, Health and Biosecurity, Canberra, ACT 2601, Australia; nias.peng@csiro.au (N.Y.G.P.); robyn.hall@csiro.au (R.N.H.); nina.huang@csiro.au (N.H.); hugh.mason@csiro.au (H.M.); ina.smith@csiro.au (I.L.S.); tanja.strive@csiro.au (T.S.); 2 Centre for Invasive Species Solutions, Bruce, ACT 2617, Australia; peter.west@dpi.nsw.gov.au (P.W.); andrew.j.read@dpi.nsw.gov.au (A.J.R.); kandarp.patel@adelaide.edu.au (K.K.P.); patrick.taggart@bushheritage.org.au (P.L.T.); 3 Ausvet Pty Ltd., Canberra, ACT 2617, Australia; 4 Vertebrate Pest Research Unit, NSW Department of Primary Industries, Orange, NSW 2880, Australia; tarnya.cox@dpi.nsw.gov.au; 5 School of Medical Sciences, The University of Sydney, Sydney, NSW 2050, Australia; jackie.mahar@csiro.au; 6 Commonwealth Scientific and Industrial Research Organisation, Australian Animal Health Laboratory and Health and Biosecurity, Geelong, VIC 3220, Australia; 7 Department of Primary Industries and Regional Development WA, Albany, WA 6630, Australia; susan.campbell@dpird.wa.gov.au; 8 Elizabeth Macarthur Agricultural Institute, NSW Department of Primary Industries, Menangle, NSW 2568, Australia; 9 Invasive Species Unit, Department of Primary Industries and Regions SA, Urrbrae, SA 5064, Australia; 10 School of Animal and Veterinary Sciences, The University of Adelaide, Roseworthy, SA 5371, Australia; 11 Vertebrate Pest Research Unit, NSW Department of Primary Industries, Queanbeyan, NSW 2620, Australia

**Keywords:** RHDV, molecular epidemiology, calicivirus, citizen science

## Abstract

Australia has multiple lagoviruses with differing pathogenicity. The circulation of these viruses was traditionally determined through opportunistic sampling events. In the lead up to the nationwide release of RHDVa-K5 (GI.1aP-GI.1a) in 2017, an existing citizen science program, RabbitScan, was augmented to allow members of the public to submit samples collected from dead leporids for lagovirus testing. This study describes the information obtained from the increased number of leporid samples received between 2015 and 2022 and focuses on the recent epidemiological interactions and evolutionary trajectory of circulating lagoviruses in Australia between October 2020 and December 2022. A total of 2771 samples were tested from January 2015 to December 2022, of which 1643 were lagovirus-positive. Notable changes in the distribution of lagovirus variants were observed, predominantly in Western Australia, where RHDV2-4c (GI.4cP-GI.2) was detected again in 2021 after initially being reported to be present in 2018. Interestingly, we found evidence that the deliberately released RHDVa-K5 was able to establish and circulate in wild rabbit populations in WA. Overall, the incorporation of citizen science approaches proved to be a cost-efficient method to increase the sampling area and enable an in-depth analysis of lagovirus distribution, genetic diversity, and interactions. The maintenance of such programs is essential to enable continued investigations of the critical parameters affecting the biocontrol of feral rabbit populations in Australia, as well as to enable the detection of any potential future incursions.

## 1. Introduction

In Australia, feral rabbits are one of the most destructive invasive pest species, costing the agricultural industry alone around AUD 239 million per year [1,2,3,4,5,6,7]. To control feral rabbit populations in Australia, rabbit haemorrhagic disease virus (RHDV) is used as a biocontrol agent. RHDV is highly contagious, causing the rapid onset of severe necrotic hepatitis, with case fatality rates of >90% among infected European rabbits (*Oryctolagus cuniculus*) [8,9]. RHDV is a non-enveloped, positive-sense single-stranded RNA (+ssRNA) virus belonging to the genus *Lagovirus* within the family *Caliciviridae* which exclusively infects leporids (rabbits and hares) [10]. Lagoviruses contain an RNA genome of approximately 7.5 kb, with two open reading frames (ORF) flanked by untranslated regions (UTRs) at both the 5′ and 3′ ends. The first ORF (ORF1) is translated into a single polyprotein that is cleaved into seven nonstructural proteins—p16, p23, 2C-like RNA helicase, p29, viral genome-linked protein (VPg), 3C-like protease (Pro), and RNA-dependent RNA polymerase (RdRp)—and one structural protein: VP60, the major capsid protein [10,11,12]. The second ORF translates to a minor structural capsid protein, VP10, that is critical for the release of the viral genome into a host cell’s cytoplasm and the production of infectious virions [13,14,15]. Lagoviruses also encode a ∼2.5 kb long subgenomic RNA (sgRNA) containing both VP60 and VP10 capsid proteins [16,17]. The viral genomic RNA (gRNA) and sgRNA are covalently linked at the 5′-UTR to VPg and polyadenylated at the 3′-UTR [10,11,12].

Due to the homology at the 5′ and 3′ ends of the gRNA and sgRNA, recombination often occurs among lagoviruses at the highly conserved breakpoint between the non-structural RdRp and structural VP60 genes [17,18]. Recombination occurs between different genogroups and/or genotypes of lagoviruses in co-infected cells, which produces recombinant lagovirus variants containing novel pairs of non-structural and structural genes. This increases virus diversity, which can improve host pathogenesis and transmissibility [19]. The nomenclature concordance of lagoviruses are classified by the type of RdRp (if known), followed by the type of VP60, in the order of genogroups, genotypes, and variants (e.g., a recombinant with genotype I.2 VP60 and genotype I.1 variant b RdRp is classified as GI.1bP-GI.2, where the “GI” denotes the genogroup, the number after the period denotes the genotype, the “b” denotes a specific variant, and the “P” denotes that the preceding genotype refers to the RdRp type) [20]. Four different lagovirus recombinants have been detected in Australia so far, some of which are reported to have emerged multiple times: RHDVa-Aus (GI.4eP-GI.1a), RHDV2 (GI.1bP-GI.2), RHDV2-4c (GI.4cP-GI.2), and RHDV2-4e (GI.4eP-GI.2) [19,21,22]. Among these recombinant lagoviruses, RHDVa-Aus and RHDV2 are exotic incursions into Australia, while RHDV2-4c and RHDV2-4e emerged locally, with the former now being the most dominant lagovirus variant detected [19,21,22].

There are currently two RHDV variants approved for use as biocontrol agents in Australia: RHDV1-V351 Czech variant (GI.1cP-GI.1c) [23,24,25,26] and RHDVa-K5 variant (GI.1aP-GI.1a) [3]. Prior to 2015, the effectiveness of RHDV1-V351 in suppressing feral rabbit populations and understanding of lagovirus epidemiology in Australia was determined through opportunistic sampling events, with less than 30 samples tested per year between 2007 and 2014 on average [24,25,26,27,28]. Although the number of samples received for lagovirus testing through opportunistic sampling events have enabled the detection of one exotic lagovirus incursion (GI.4eP-GI.1a) in 2014, it remained inadequate in providing a rigorous understanding of lagovirus epidemiology and associated fluctuations in Australia due to a lack of sufficient samples received for periodic lagovirus testing [29].

In the lead up to the national release of the RHDVa-K5 biocontrol agent in 2017, an existing citizen science platform, “RabbitScan” (https://www.feralscan.org.au/rabbitscan/, accessed on 23 October 2023), was augmented to improve the surveillance of lagovirus distribution and feral rabbit management at a national scale by providing members of the public an option to request a sampling kit to submit tissue samples from deceased wild and domesticated leporids for lagovirus testing [3,30]. Previously, RabbitScan only allowed citizens to contribute to the monitoring of feral rabbit distribution and abundance by reporting rabbit sightings, rabbit-associated ecological damages such as soil erosion, and control activities. In addition to RabbitScan, rabbit tissue samples could also be submitted directly to the Commonwealth Scientific and Industrial research Organisation (CSIRO) rabbit biocontrol team (https://research.csiro.au/rhdv/testing/, accessed on 23 October 2023) for testing.

Citizen science is now regularly employed in multiple disciplines, such as biological conservation [31], invasive species management [32,33,34,35,36], and education [37], substantially impacting data collection. It has enabled researchers to obtain large datasets with sample numbers ranging from the tens to the millions, depending on the scale of the population and geography by which the citizen science program is implemented. Citizen science was incorporated to consistently improve the monitoring of lagovirus epidemiology, following the nationwide release of RHDVa-K5 in 2017, and enable us to study potential lagovirus interactions and epidemiological changes as multiple competing co-circulated variants. Furthermore, the improved understanding of lagovirus molecular epidemiology could help to better inform the deployment of RHDVa-K5 and thus improve its effectiveness in suppressing feral rabbit populations.

Here, we elaborate on the types of information obtained from the increased number of leporid samples received via citizen science between January 2015 and December 2022 and focus on the recent molecular epidemiology and evolutionary trajectory of circulating lagoviruses in Australia from October 2020 to December 2022. Furthermore, we describe the sustained circulation of RHDVa-K5 in Western Australia.

## 2. Methods

### 2.1. Sample Collection and RNA Extraction

Samples collected from dead rabbits and hares between January 2015 and December 2022 from our citizen science-driven lagovirus surveillance program were used in this study to determine the molecular epidemiology and evolutionary trajectory of lagoviruses in Australia between 2020 and 2022. This includes previously described leporid samples (rabbits and hares) collected from 2016 to 2020, which were included in this work for analysis purposes [19]. No animal ethics approvals are required for sampling rabbits that are found dead in Australia. Samples from deceased wild and domesticated leporids were submitted through either our online citizen science platform, RabbitScan (https://www.feralscan.org.au/rabbitscan/, accessed on 23 October 2023), or directly to the CSIRO rabbit biocontrol team for lagovirus testing (https://research.csiro.au/rhdv/testing/, accessed on 23 October 2023). In both cases, members of the public were provided submission kits and instructions to collect and store samples for delivery to our testing laboratory located in Canberra, ACT, Australia. Citizen scientists were instructed to harvest part of the liver tissue (as a first preference) or bone marrow and other tissue samples if the liver tissue was inaccessible. Tissue samples were provided either fresh and frozen or stored in an RNA stabilization solution [38]. Lagovirus-positive diagnostic samples tested at the NSW state veterinary laboratory were also referred to the CSIRO to augment the community submissions. RNA from tissue samples (liver and bone marrow) was extracted using the Maxwell^®^ RSC instrument (Promega, Madison, WI, USA) in combination with the Maxwell^®^ RSC SimplyRNA Tissue Kit (Promega) or the RNeasy mini kit (Qiagen, Clayton, VIC, Australia) according to the manufacturer’s instructions. Prior to extraction, the tissue samples were homogenized using glass beads and a Precellys 24-dual tissue homogenizer (Bertin Technologies, Montigny-le-Bretonneux, France).

### 2.2. RT-qPCR and Variant Identification

Samples were first screened for the presence of lagoviruses using a universal SYBR-green-based RT-qPCR assay as previously described [22]. Positive samples were subjected to a multiplex RT-PCR assay to determine the genotype as described previously [22,39]. Samples positive for RHDV2 (GI.2) were further analysed to distinguish between the different variants. A RT-PCR spanning the RdRp-VP60 junction was carried out to generate an amplicon for verification via Sanger sequencing as described previously [40].

### 2.3. Sequencing

A selection of positive samples (3 samples per genotypic variant for each yearly quarter per state/territory if possible) were subjected to whole-genome sequencing to determine the molecular epidemiology and evolution of lagoviruses in Australia. Samples were selected with a preference for wild rabbits to best cover the rabbit lagovirus landscape in Australia. Extracted RNA underwent reverse transcription using SuperScript IV reverse transcriptase (Invitrogen, Waltham, MA, USA) using a ProFlex PCR Thermocycler System (Applied Biosystems, Waltham, MA, USA). Whole genomes were amplified from synthesized cDNA in overlapping fragments (primers listed in Table S1) and the Platinum™ Taq DNA Polymerase High Fidelity Kit (Invitrogen). The amplified products were detected using a 1% Agarose gel stained with SYBR™ Safe DNA Gel Stain (Invitrogen). PCR products were subsequently purified using AMPure XP Magnetic Beads (Beckman Coulter, Lane Cove West, NSW, Australia) and quantified using the Qubit™ 1X dsDNA High Sensitivity (HS) Assay Kits (Invitrogen) or Quant-iT™ dsDNA Assay Kits, high sensitivity (HS) (Invitrogen). Library preparation was performed using the Nextera XT DNA Library Preparation Kit (Illumina, Melbourne, VIC, Australia) as previously described [19]. The quality of the generated libraries was assessed using the Agilent 2200 TapeStation System and D1000 high-sensitivity tapes (Agilent Technologies, Mulgrave, VIC, Australia). Sequencing was performed on an Illumina MiSeq instrument (300 cycles, v2).

### 2.4. Sequence Data Analysis

The quality of the sequence data was assessed using FastQC (v0.11.08), and adapter, primer, and low-quality sequences were subsequently trimmed using Trimmomatic [41] as previously described [38]. Cleaned reads were then mapped against the lagovirus GI reference genome (Accession number M67473.1) in Geneious Prime 2022.2.1 to generate consensus sequences. A number of previously reported lagovirus sequences obtained from samples acquired up until 2018 were included in this study [19,21,22,29,38,39] to determine the genomic epidemiology and evolution of lagoviruses in Australia from January 2007 to December 2022.

### 2.5. Bayesian Evolutionary Analyses

All available RHDV2-4c sequences (n = 169) were aligned using MAFFT (v7.490) [42]. Sequences were trimmed by removing the 3′- and 5′-UTRs. As these viruses are recombinants, with the breakpoint between the non-structural and structural genes, the alignments were split into non-structural genes (upstream of the recombination breakpoint) and structural genes (downstream of the recombination breakpoint). The alignments of structural (S) and non-structural (NS) genes were screened using TempEst [43] to confirm sufficient temporal signal. The strongest temporal signal (correlation coefficient = 0.9257) was found for the NS alignment compared to the S alignment (correlation coefficient = 0.8022). Accordingly, the NS genes alignment was used for all further analyses. A Bayesian Markov chain Monte Carlo (MCMC) approach was used to infer a time-scaled phylogeny. Marginal likelihood estimations (MLE), as implemented in BEAUti (v1.10.4), were used to assess the most appropriate clock prior (strict versus uncorrelated log-normally distributed (UCLD)) and tree prior (Gaussian Markov random field Bayesian skyride model versus constant size coalescent versus exponential coalescent). The (GTR+F+I+G4) substitution model, as determined using Modeltest implemented in IQ-Tree (v2.2.0.5) [44], was used for each analysis. The best MLE values were reached using a UCLD clock prior and the Bayesian skyride model and were used for a subsequent Bayesian Evolutionary Analysis Sampling Tree (BEAST) (v1.10.4) [45]. To confirm consistency, the analysis was run twice to convergence (ESS > 200).

### 2.6. Data Visualization

All figures were plotted in RStudio (v4.2.2) using packages ggplot2 (v3.4.2) [46], tidyverse (v2.0.0) [47], dplyr (v1.1.1) [48], readxl (v1.4.2) [49], svglite (v2.1.1) [50], ggpubr (v0.6.0) [51], RColorBrewer (v1.1-3) [52], rgdal (v1.6-5) [53], sf (v1.0-1.2) [54], shapefiles (v0.7.2) [55], terra (v1.7-23) [56], raster (v3.6-20) [57], ggthemes (v4.2.4) [58], ggtree (v.3.8.0) [59], and treeio (v.1.24.1) [60].

## 3. Results

### 3.1. The Genomic Epidemiology of Lagoviruses Is Improved through Citizen Science

**Figure 1:**
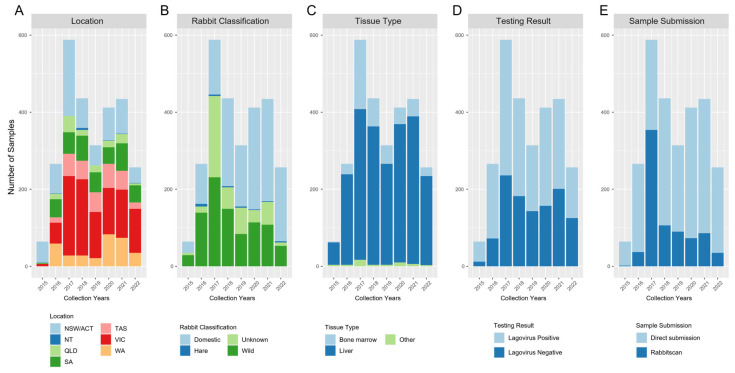
An overview of the total leporid samples surveyed from 2015 to 2022 using citizen science via direct sample submission or the RabbitScan app (presented in the form of a stacked bar chart). The samples were categorized annually into (**A**) the state of the sampling location (NSW—New South Wales, VIC—Victoria, QLD—Queensland, NT—Northern Territory, WA—Western Australia, SA—South Australia, ACT—Australian Capital Territory, TAS—Tasmania), (**B**) the types of leporid (domestic rabbits, wild rabbits, hares, and unknown rabbits), (**C**) the type of tissue used for testing, (**D**) the number of positive and negative results, and (**E**) the sample submission pathway. Individual colours are assigned to each variable within the subplots.

**Figure 2:**
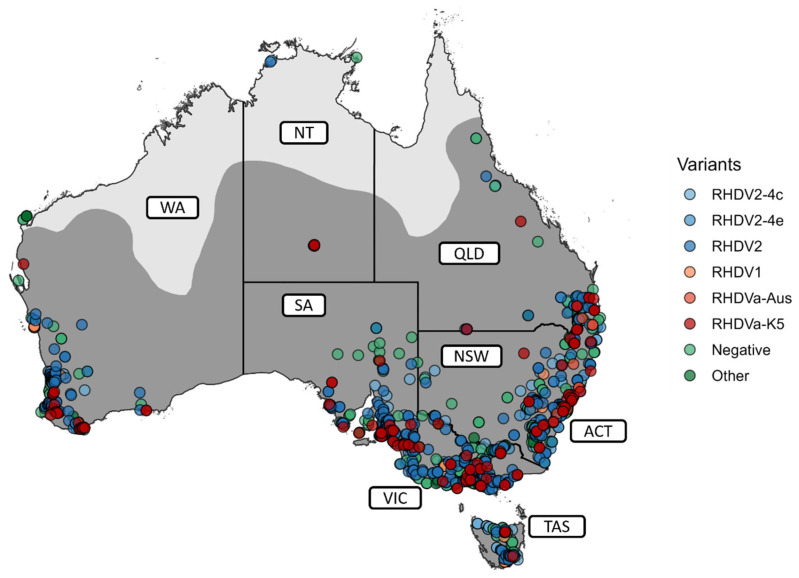
Geographical distribution of rabbit population and lagovirus-positive samples from January 2015 to December 2022 in Australia. Rabbit population distribution was determined from a previous study and is depicted as a dark grey area in the map [61]. Colours refer to the respective variants detected. “Other” refers to samples that tested negative for RHDV but positive for myxomavirus, *Pasteurella multocida*, or *Eimeria* spp. NSW—New South Wales, VIC—Victoria, QLD—Queensland, NT—Northern Territory, WA—Western Australia, SA—South Australia, ACT—Australian Capital Territory, TAS—Tasmania.

In the period in which the citizen science-driven lagovirus surveillance program was introduced, from January 2015 to December 2022, a total of 2771 samples were submitted and analysed for lagoviruses with the help of citizen scientists (Figure 1). In detail, rabbit tissue samples were received throughout Australia—from the Australian Capital Territory (ACT) and New South Wales (NSW, ACT/NSW n = 674), Victoria (VIC, n = 943), South Australia (SA, n = 380), Queensland (QLD, n = 136), Western Australia (WA, n = 328), Tasmania (TAS, n = 300), and the Northern Territory (NT, n = 10) (Figure 1A and Figure 2).

Most samples were collected from domestic rabbits (n = 1383) compared to wild rabbits (n = 907) and hares (n = 30) (Figure 1B). The proportion of sample sources between wild and domestic rabbits changed in 2018. While most samples were of wild origin from 2015 to 2017, this trend switched, and from 2018, domesticated rabbit samples constituted the majority of the samples. A considerable number of samples (n = 457), especially in 2017 (n = 211), also came from rabbits of unknown origin (labelled as “Unknown” in Figure 1B). This meant that no information was provided upon receiving the sample on whether the sample was from a domestic or wild rabbit. However, these samples were likely from wild rabbits, as these samples were submitted during the RHDVa-K5 release program and through the RabbitScan app. The types of submitted samples were mostly liver (n = 2278), followed by bone marrow (n = 441) and other tissues such as blood, kidney, and maggots from carcasses (“Other”, n = 52) (Figure 1C). Out of the 2771 samples tested between January 2015 and December 2022, 1643 (59.29%) were lagovirus-positive, while 1076 (38.83%) samples were negative (Figure 1D). Samples were mostly submitted directly to CSIRO, except in 2017, when the majority of samples were received through the RabbitScan app, associated with the large engagement program rolled out to landholders associated with the RHDVa-K5 release at this time (Figure 1E).

### 3.2. The Diversity of Lagoviruses Has Been Severely Reduced with the Emergence of RHDV2-4c

**Figure 3:**
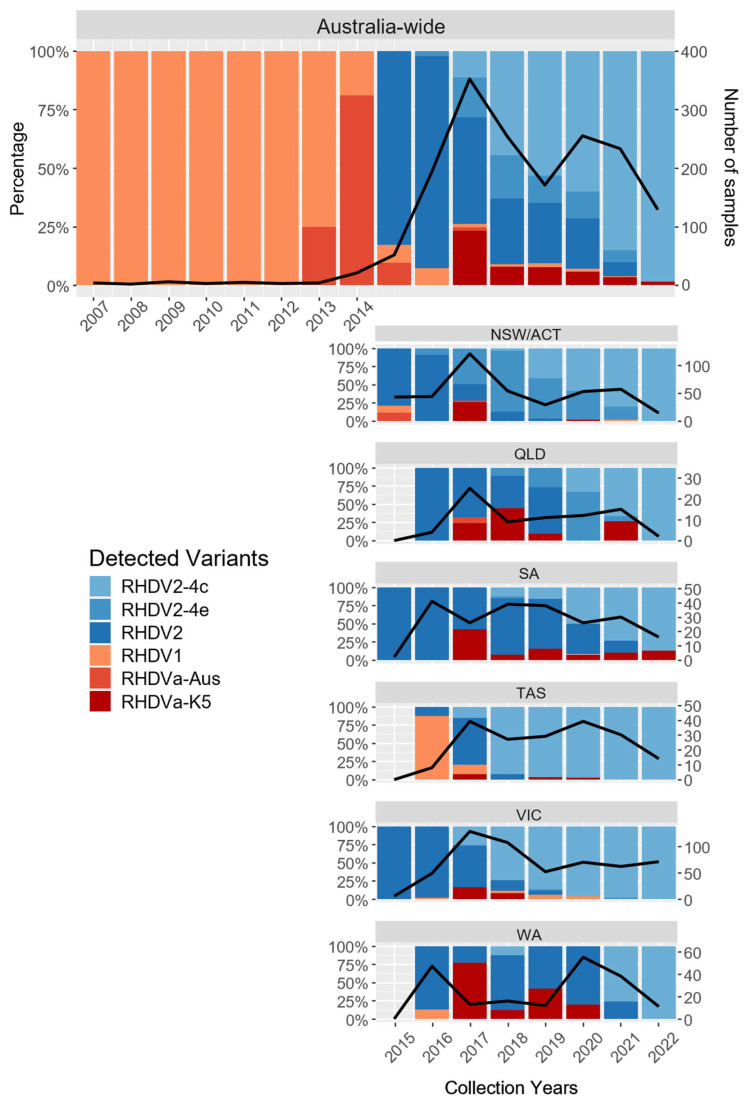
Distribution of pathogenic GI lagovirus variants detected in Australia from 2007 to 2022. The Australia-wide barplot represents the percentage of each variant of lagovirus-positive samples from 2007 to 2022 in Australia. The black line represents the total number of samples analysed. The nationwide lagovirus distribution bar plot is further facetted by state and territory from 2015 to 2022 in relation to the introduction of the citizen science-driven lagovirus surveillance program. The respective lagovirus variants are differentiated by their individual colours. Results for the Northern Territory are not shown due to low numbers. NSW—New South Wales, VIC—Victoria, QLD—Queensland, WA—Western Australia, SA—South Australia, ACT—Australian Capital Territory, TAS—Tasmania.

Among the lagovirus-positive samples collected between January 2015 to December 2022, most were RHDV2-4c (GI.4cP-GI.2; n = 722), followed by RHDV2 (GI.1bP-GI.2; n = 563), RHDV2-4e (GI.4eP-GI.2; n = 172), RHDVa-K5 (GI.1aP-GI.1a; n = 140), RHDV1 (GI.1c; n = 33), and RHDVa-Aus (GI.1a; n = 10) (Figure 2 and Figure 3). After 2020, the diversity of lagoviruses remained largely unchanged, except for the absence of RHDVa-Aus among the RHDV-positive samples (n = 1042). From 2020 to 2021, the overall frequencies of RHDV2-4e, RHDV2, RHDV1, and RHDVa-K5 declined. In contrast, the frequency of RHDV2-4c across Australia rose to 84.97% (n = 198/233), and by December 2022, RHDV2-4c had completely replaced RHDV2-4e and RHDV2 in Australia (98.49%, n = 130/132), with the only other variant detected being RHDVa-K5 (n = 2) in SA (Figure 3).

The submitted samples were mostly from the major cities or densely populated areas of Australia (Figure S1), despite the widespread distribution of feral rabbit populations across Australia (Figure 2) [61]. As expected, RHDVa-K5 was detected at locations where it was periodically released to aid the control of local wild rabbit populations (Figure 2). While at least six recombination events between different lagoviruses occurred between January 2014 and September 2020 [19], no further recombination events among lagoviruses in Australia were observed between October 2020 and December 2022 (Figure 3).

### 3.3. The Delayed Emergence of RHDV2-4c in Western Australia

Our previous study on the emergence of RHDV2-4c recombinants (first detected in VIC in early 2017) in Australia revealed five distinct lineages (lineages i–iv), each of which emerged through independent recombination events [19]. To further analyse the spread, especially for WA, we included new RHDV2-4c sequences (n = 71) obtained from every state in Australia between 2020 and 2022 into the existing dataset. All available positive RHDV2-4c samples from WA since the first occurrence in 2018 were also included in the analysis.

**Figure 4:**
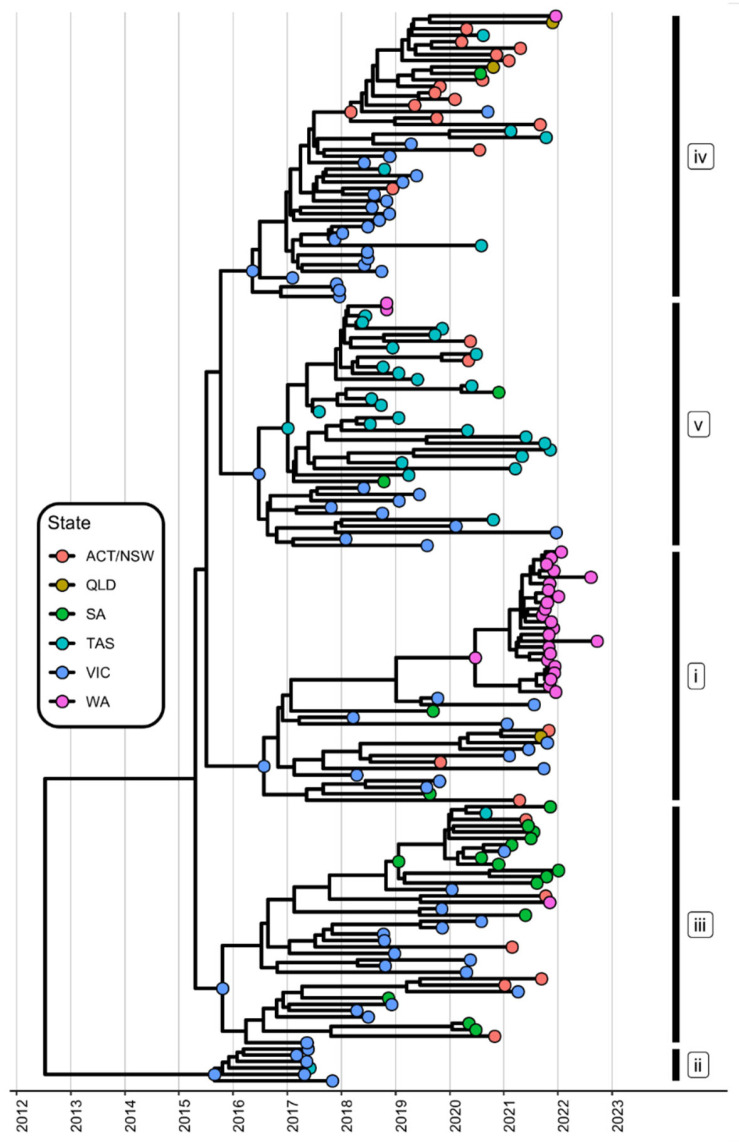
A time-structured phylogenetic analysis of Australian RHDV2-4c non-structural gene sequences. Tip colours and points at internal nodes represent the respective states of sampling (terminal nodes) or the most likely ancestral state (internal nodes), as calculated by ancestral state reconstruction. Clades are labelled by lineage (i–iv). Ancestral state reconstruction is only depicted for internal nodes at the emergence of new lineages or subclades within lineages. Results for the Northern Territory are not shown due to low sample numbers. NSW—New South Wales, VIC—Victoria, QLD—Queensland, WA—Western Australia, SA—South Australia, ACT—Australian Capital Territory, TAS—Tasmania.

Interestingly, all recombinant lineages were detected in VIC at least once. However, not all five lineages continue to exist, as lineage ii has not been detected throughout Australia since 2017. So far, no recombinant lineage has established widely in QLD, with only sporadic detections being reported. Considering the sparse sampling in QLD (Figure 1 and Figure 2), this could be a result of sampling bias. From late 2021 onwards, we found evidence of RHDV2-4c establishment in Western Australia (lineage i) (Figure 4). Interestingly, RHDV2-4c was first detected in WA in 2018 [19]. This single introduction was identified to be lineage v but did not circulate in WA beyond the initial detection. This was most likely because it was detected in two domestic breeding stock rabbits imported to WA from TAS without further opportunity for transmission due to a lack of exposure to naïve wild rabbit populations and proper biosecurity measurements [19]. No further RHDV2-4c variants were detected in WA until 2021 (Figure 4). In 2021, RHDV2-4c from lineages i (n = 23), iii (n = 2), and iv (n = 1) was detected in positive samples, but only lineage i has successfully become established in WA (Figure 4). Ancestral reconstruction suggests that lineages i and iii originated from VIC, while lineage iv seemed to have originated from NSW/ACT. No lineage v RHDV2-4c samples have been detected in WA since 2017, suggesting a potential extinction of lineage v in WA, while the identification of lineages iii and iv was more recent, and these lineages may still become established in the future.

### 3.4. The Circulation of RHDVa-K5 in Western Australia Following Releases

RHDVa-K5 had limited success spreading beyond its release sites throughout eastern Australia (Figure 3). In 2017, the year it was first released, RHDVa-K5 constituted 23.3% of the positive samples, likely because of increased sampling surrounding the release sites (Figure 3). An analysis of the epidemiological metadata submitted with RHDVa-K5-positive samples revealed that all samples were reported to be associated with recent releases. Although RHDVa-K5 was detected on a regular basis in sample submissions (from around release sites), it was ultimately outcompeted by RHDV2 (GI.1bP-GI.2) and the recombinant variants RHDV2-4c and RHDV2-4e from 2018 onwards (Figure 3). Excluding WA, this provisionally suggests that RHDVa-K5 did not circulate beyond its release sites and that selective sampling following localized releases likely decreased in later years.

Increasing numbers of RHDVa-K5-positive samples (2019, n = 5; 2020, n = 11) were reported from locations within WA that were not associated with recent virus releases following a drop in detections in 2018 (n = 2). To further investigate this unexpected increase in RHDVa-K5 cases, we analysed and quantified the number of single-nucleotide polymorphisms (SNPs) present in those circulating RHDVa-K5 whole-genome virus sequences and compared them to the number of SNPs from sequences of RHDVa-K5 from known release sites. Lagoviruses, as +ssRNA viruses, have extraordinarily high evolutionary rates [38,39]; therefore, the number of SNPs is expected to accumulate among lagoviruses within a few generations of circulation in the natural host.

**Figure 5:**
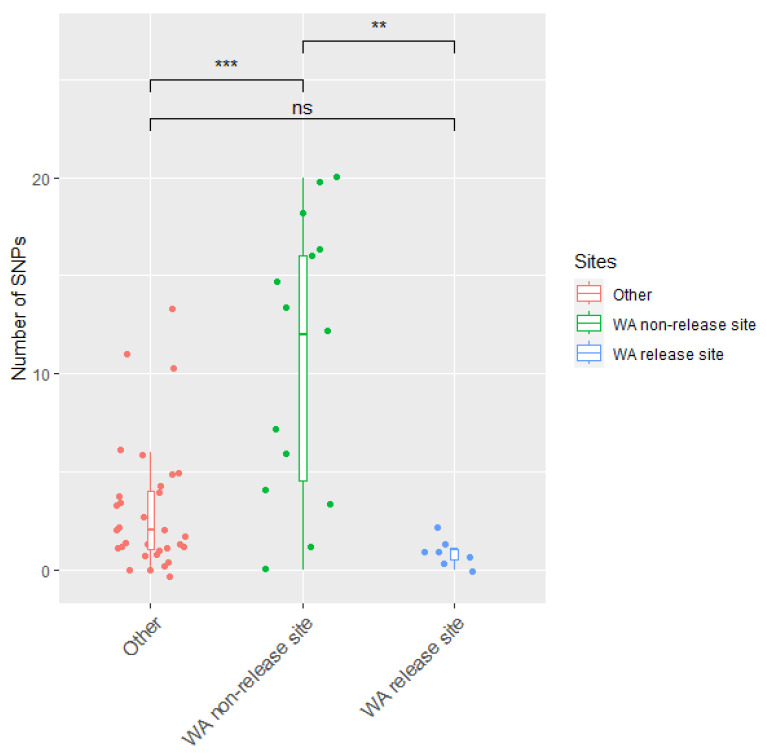
The number of single-nucleotide polymorphisms (SNPs) from WA release sites, non-release sites, and all other detections of RHDVa-K5 in Australia. Scatter box plots were added to show the median, and outliers are represented as dots within the violin plot. Significance levels of *t*-tests are shown as asterisks (*). **: p ≤ 0.01, ***: p ≤ 0.001, ns: *p* > 0.05 (not significant). WA—Western Australia.

**Figure 6:**
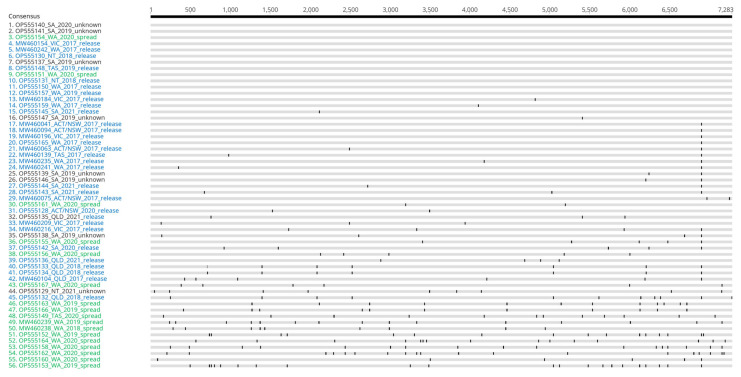
Genome alignment of all RHDVa-K5 isolates comparing the number of SNPs accumulated between those from release sites versus non-release sites. Isolates are coloured to distinguish between RHDVa-K5 isolates from release sites (blue) or non-release sites (green). RHDVa-K5 isolates from undetermined released or non-released sites are coloured black.

Our analysis showed that viral genomes from non-release sites in WA harboured significantly more SNPs compared to those from release sites in WA and to RHDVa-K5 sequences from the rest of Australia (Figure 5 and Figure 6). While the median number of SNPs for the release sites in WA and the rest of Australia was 1 and 2, respectively, this median value increased to 12 for samples from non-release sites in WA (Figure 5 and Figure 6). These findings suggest the continued circulation of RHDVa-K5 in WA, since more SNPs were accumulated as these viruses circulated from host to host.

## 4. Discussion

The combination of citizen science sampling through RabbitScan and direct rabbit sample submission to CSIRO for lagovirus testing substantially increased sampling numbers and improved lagovirus surveillance in Australia compared to either sampling approach alone. Notably, it enabled the early detection of an exotic incursion of RHDV2 (GI.1bP-GI.2) into Australia in 2015 [21,39]. Additionally, it allowed for subsequent monitoring of the evolutionary trajectory of lagoviruses in Australia, such as the detection of recombination events, which generated six recombinant variants between RHDV2 and a benign non-pathogenic lagovirus (GI.4)—RHDV2-4e (GI.4eP-GI.2) and RHDV2-4c (GI.4cP-GI.2) between 2015 and 2017 [19,22].

Biocontrol agents such as RHDV are a key tool for managing rabbit populations at the landscape scale in Australia. As such, a thorough understanding of the spread, diversity, interactions, and evolution of these viruses is critical to support the development of future control strategies. Furthermore, the timely detection of events that may affect the effectiveness of these viruses as suppression tools, such as the emergence of novel RHDV recombinants or the extinction of RHDV lineages, is essential.

Prior to 2015, no systematic, Australia-wide molecular analysis of lagoviruses was conducted. This resulted in a limited understanding of the genetic diversity of lagoviruses and their distribution in Australia. The addition of a sample submission feature into RabbitScan [3] and direct submission to CSIRO allowed for the broadscale submission of samples from deceased wild and domesticated leporids for lagovirus testing throughout the year. This expanded sampling networks, increasing submissions from 30 to an average of 345 samples per year over an 8-year period between 2015 and 2022, thereby improving the surveillance of lagovirus epidemiology in Australia.

An intriguing observation from our study was the change in the type of samples submitted for lagovirus testing over the years. At the beginning of the program, and during the nationwide release of RHDVa-K5, the majority of samples originated from wild rabbits or from rabbits of unknown origin. This trend shifted after the official release program ended, and the numbers of samples were skewed towards domestic rabbits from 2018 (Figure 1B). The number of wild rabbit sample submissions decreased in the years after the release of RHDVa-K5, possibly due to an overall decrease in interest towards the program, with a reduction in reminders to citizens to submit samples for testing after the end of the release project as well as a significant decrease in wild rabbit populations and sightings due to the continued circulation of RHDV2 rather than a lowered effectiveness of the release biocontrol virus [3,62]. In contrast, the number of domestic rabbit sample submissions has remained stable since the release, as pet owners remain motivated to find out whether lagoviruses are responsible for the death of their animals. Although our data on the molecular epidemiology of lagoviruses consist of more domestic rabbit samples than wild rabbit samples, domestic rabbits are considered appropriate sentinels for circulating lagovirus variants. Domestic rabbits are likely to acquire RHDVs through contaminated rabbit feeds, items, or insects (flies) that were previously in contact with infected carcasses of wild rabbits, since RHDV can remain infectious in the environment for a minimum of 5 to 20 days [63,64]. This is further evidenced by the fact that RHDV sequences obtained from domestic rabbit samples cluster closely among virus sequences from wild rabbits, irrespective of variant (Figure S2), highlighting that domestic rabbit samples serve as good indicators of the natural circulation of lagoviruses in the wild. Another trend was that most samples were submitted from regions with higher human population densities (Figure S1) [65]. While this is not unexpected, it makes it harder to estimate the spread and impact of RHDV in remote areas. The only approach to reduce these challenges is with targeted surveillance efforts in remote areas, which are time- and cost-intensive.

Consistent with what was observed up until 2020 in a previous study [19], this study found that RHDV2-4c (GI.4c-GI.2) continues to be the dominant lagovirus variant in European rabbits and hares in Australia (as of December 2022) since its emergence in VIC in 2017. Throughout 2022 (the last year sampled for this study), no variants other than RHDVa-K5 were detected. Moreover, we continued to observe that there were no signs of dominance between one lineage of RHDV2-4c over another across Australia (except for lineage, ii which appears to have died out). This suggests that (i) there are no apparent fitness advantages of one RHDV2-4c lineage over another and that (ii) the incursion of different lineages in different states is likely due to founder effects, in which the early establishment and subsequent circulation of virus lineages were stochastic. This is likely because, genotypically, the five RHDV2-4c lineages remained the same despite having evolved through five separate lineages from independent recombination events. Furthermore, the early establishment of these RHDV2-4c lineages and the rapid displacement of other lagoviruses, such as RHDV2 (GI.1bP-GI.2) in SA and VIC and RHDV2-4e (GI.4eP-GI.2) in NSW/ACT, could also be strongly promoted through opportunistic infections of larger number of rabbits from rabbit colonies in the wild or in commercial rabbitries, leading to a higher probability of virus dissemination. Overall, the addition of our data provides an up-to-date understanding of the genomic epidemiology and evolutionary trajectory of lagoviruses in Australia.

An interesting finding is the spread of RHDV2-4c into WA. As previously described, RHDV2-4c was first detected in WA in 2018 from a single interstate incursion, but no further RHDV2-4c was detected in WA until 2021 [19]. In 2021, RHDV2-4c was introduced to WA on at least three different occasions, each from a different lineage, but only the incursion of lineage i managed to spread and establish in WA, according to contemporary sequencing efforts. The ancestor variant of this lineage originated in Victoria; however, it is unclear how the virus spread. It is most likely that it was transported across the country through pet rabbit movements or the movement of contaminated items, as VIC and WA are not neighbouring states, and the phylogenetic analysis did not support a spread through SA (the state in between). So far, all generated sequences within this lineage are derived from domesticated pet rabbits, which represent an indirect conduit for transmitting lagoviruses, as deceased pet rabbits are often placed in compost heaps and buried in soil. This is easily accessible to scavengers like feral cats and foxes and flies, which will then facilitate further lagovirus transmission upon exposure. Additionally, other pet rabbits could also be infected with lagoviruses through contact with contaminated cages for transportation or housing. The only RHDV2-4c sequence from wild rabbits recovered so far in WA belonged to lineage iv and was most closely related to a sequence recovered from ACT/NSW. We hypothesize that human interventions, such as transporting pet rabbits, rabbit-housing cages, or contaminated items, have facilitated virus migration from east to west across Australia.

Another finding of note is the confirmation of the establishment of RHDVa-K5 in wild rabbits in WA. After the national release program in 2017, RHDVa-K5 failed to establish among wild rabbit populations in any other state or territory. It is feasible that the late arrival and establishment of RHDV2-4c in WA may have helped with the establishment of RHDVa-K5 in wild rabbits. The initial incursion of RHDV2 into WA likely first displaced RHDV1-V351 from the lagovirus population, as previously observed in other states [3,39]. The eastern states faced a rapid spread of RHDV2 incursion that diminished wild rabbit populations before the release of RHDVa-K5, and consequently, likely hindered the circulation and establishment of RHDVa-K5. An analysis of the SNPs in viral genomes recovered from RHDVa-K5-positive cases around the country, together with the increased detection of RHDVa-K5-positive samples, support the hypothesis that there was a low-level circulation of RHDVa-K5 in WA before the establishment of RHDV2-4c in 2021. All deliberately released RHDVa-K5 preparations are derived from the same commercially produced virus stock and show little genetic variability. Therefore, in RHDVa-K5-positive cases associated with deliberate releases where rabbits have died from the baits they have consumed, the number of SNPs present is very low. The number of SNPs in WA from non-release sites reached up to 20 and was therefore significantly higher than across other Australian states or known WA release sites, indicating some level of RHDVa-K5 establishment and circulation among wild rabbits in WA. We hypothesize that this is highly influenced by the delayed arrival of RHDV2 and its recombinants into WA compared to the rest of the Australian states. This delay allowed RHDVa-K5 to circulate with less competition from other RHDV variants with higher epidemiological fitness. Hence, with the absence of RHDV2 and the subsequent emergence of RHDV recombinants with increased epidemiological fitness, RHDVa-K5 may have been able to establish more widely across Australia and possibly have a greater impact in suppressing wild rabbit populations than what was observed.

Contrary to our previous study, at least six recombination events occurred among lagoviruses between 2016 and 2017 [19], and no new recombinant lagovirus variants were detected among lagovirus-positive samples collected from 2018 onwards. Coincidentally, these recombination events occurred shortly following the incursions of RHDVa-Aus (GI.4eP-GI.1a) in 2014 and RHDV2 (GI.2) in 2015 [21,29], resulting in recombinant variant RHDV2-4e (GI.4eP-GI.2) in 2016 after recombining together, as well as in recombinant variant RHDV2-4c (GI.4cP-GI.2) in 2017 after RHDV2 recombined with an endemic benign GI.4 lagovirus [19,22]. The lack of new recombination events detected from our citizen science-enhanced sampling data suggests that all potential beneficial recombination (i.e., genetic recombination resulting in phenotypic advantages to virus fitness and host transmission) among the lagovirus population is likely to have occurred rapidly during opportunistic co-circulation among the feral rabbit populations or large domesticated rabbitries. Additionally, the presence of RHDV2 nationwide has been decreasing rapidly since 2018, which in turn, reduces the probability of co-infection with other circulating strains for new recombination to occur. Alternatively, there could have been more recombination events which gave rise to less transmissible or viable recombinant variants compared to RHDV2-4e and RHDV2-4c, which have remained undetected. It is important to note that our current diagnostic assays to detect novel lagovirus recombinants are restricted to detect recombinants with the RHDV2 GI.2 capsid. We did not attempt to design new diagnostic assays to identify potential novel recombinants outside of the RHDV2 GI.2 capsid, as existing examples of lagovirus recombination events have only been reported with the RHDV2 GI.2 capsid [18,22,66,67].

Our study did not include screening for the non-pathogenic lagoiviruses (GI.3 and GI.4) of the samples submitted by the public. This is largely because non-pathogenic lagoviruses have a different tissue tropism to their pathogenic siblings, targeting the enteric tissues (especially the duodenum) rather than the liver. Although additional sample collection and analysis of gut samples may have theoretically been possible, this was not carried out. For the detection and genetic analysis of non-pathogenic lagoviruses, young healthy rabbits (as opposed to deceased rabbits) are a better source of materials, as this is when they are most likely to contract an infection [68], and fresh samples are a more reliable source to detect lagoviruses in gut tissues that rapidly deteriorate after death. In addition, parallel studies involving the systematic collection of healthy shot rabbits and serological analysis were carried out as a more reliable means to estimate the presence and prevalence of non-pathogenic lagoviruses [62,69].

In conclusion, our augmented citizen science program was (and still is) a cost-efficient and robust approach to enabling a better understanding of the molecular epidemiology of lagoviruses. The program has enhanced the surveillance of lagovirus diversity in Australia and identified the emergence of two new RHDV2 recombinant genotypes while uncovering the true evolutionary history of the multiple recombination events that gave rise to one of these recombinant genotypes [19]. This level of evolutionary analysis was only possible because of the depth of sampling since the introduction of the citizen science program. Furthermore, we were able to accurately determine and monitor the molecular epidemiology of circulating lagoviruses in Australia. Altogether, consistent with numerous studies in the literature involving citizen science [32,33,34,35,36], the implementation of the rabbit and lagovirus surveillance program has demonstrated that it is a valuable resource to reliably obtain robust data to determine and monitor the genomic epidemiology and diversity of lagoviruses. It is therefore critical to sustain such programs for the long term to monitor the emergence of new RHDV incursions and/or recombinant variants which may affect wild and domestic rabbit populations.

## Data Availability

All generated sequences that were newly generated in this study and used for further analysis within this study were deposited in GenBank (Accession numbers: OP555128-OP555167, OP588038- OP588108, OP588237-OP588273, OP642067-OP642071, OQ414452-OQ414471).

## Supplementary Materials

The following supporting information can be downloaded at: https://www.mdpi.com/article/10.3390/v15122348/s1, Figure S1: A geographical distribution of the human population and samples submitted for lagovirus testing from January 2015 to December 2022 in Australia. The human population distribution is depicted in dark grey and was determined based on the 2021–22 population data published by the Australian Bureau of Statistics [65]. Colors refer to the respective lagovirus variant that was detected. “Other” refers to samples that tested negative for RHDV but positive for Myxomavirus, Pasteurella or Eimeria. NSW—New South Wales, VIC—Victoria, QLD—Queensland, NT—Northern Territory, WA—Western Australia, SA—South Australia, ACT—Australian Capital Territory, TAS—Tasmania; Figure S2: Organization of RHDV isolates from different sources of leporid samples tested based on non-structural gene genotype. Samples included those obtained from domesticated rabbits (red), hares (blue), unknown (purple) and wild rabbits (green). The reference sequences for GI.1 and GI.4 are colored in yellow. Respective genotypes are differentiated and highlighted: (A) GI.1 and (B) GI.4; Table S1: PCR strategy for lagovirus amplification [40,70]; Table S2: PrimalScheme PCR strategy for lagovirus amplification [19,40,70].
