# Supplementary material for: Utilizing Molecular Epidemiology and Citizen Science for the Surveillance of Lagoviruses in Australia
Source: Viruses. 2023 Nov 29;15(12):2348. doi: 10.3390/v15122348 (PMC10747141; doi:10.3390/v15122348)
Supplement: Supplementary file 1 [file viruses-15-02348-s001.zip › viruses-2706018-supplementary.pdf]

Supplementary Information

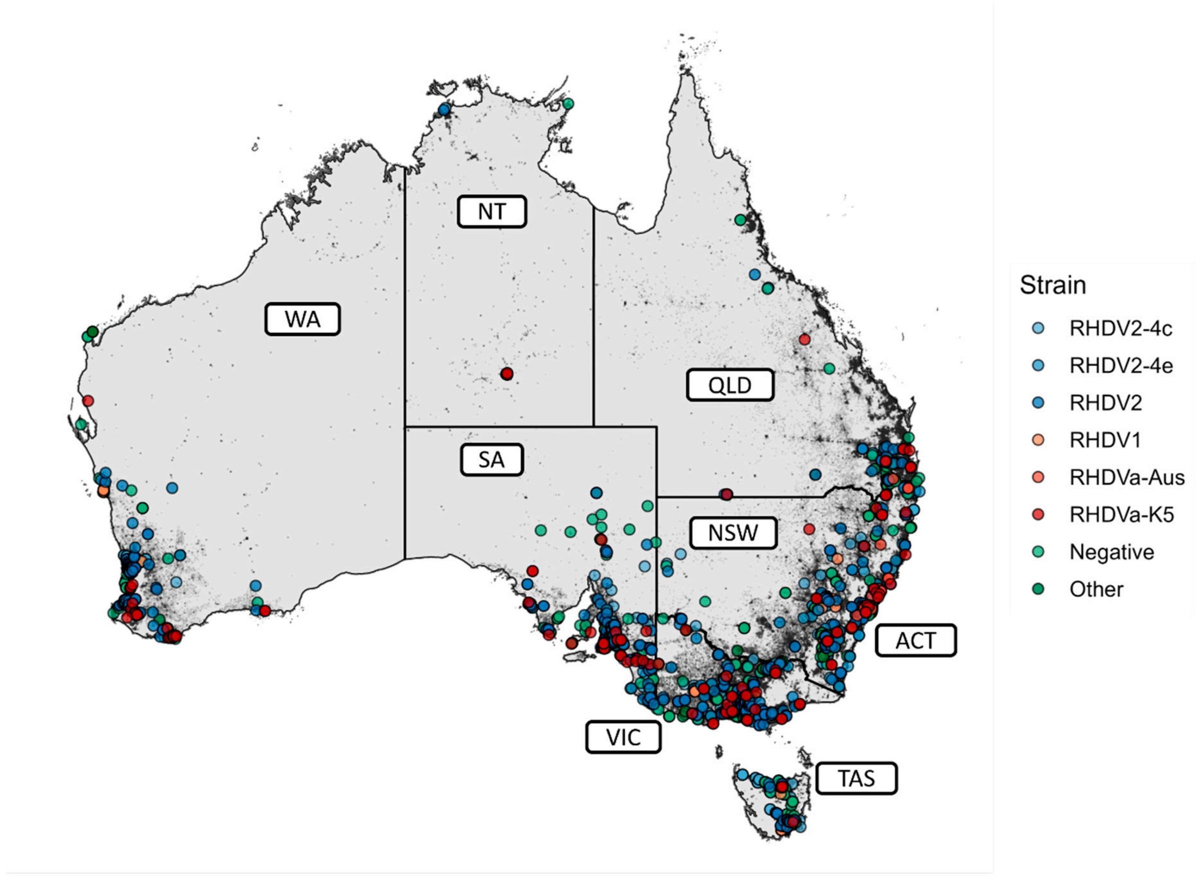

FIG S1. A geographical distribution of the human population and samples submitted for lagovirus testing from January 2015 to December 2022 in Australia. The human population distribution is depicted in dark grey and was determined based on the 2021–22 population data published by the Australian Bureau of Statistics [1]. Colours refer to the respective lagovirus variant that was detected. “Other” refers to samples that tested negative for RHDV but positive for Myxomavirus, Pasteurella or Eimeria. NSW – New South Wales, VIC – Victoria, QLD – Queensland, NT – Northern Territory, WA – Western Australia, SA – South Australia, ACT – Australian Capital Territory, TAS – Tasmania.

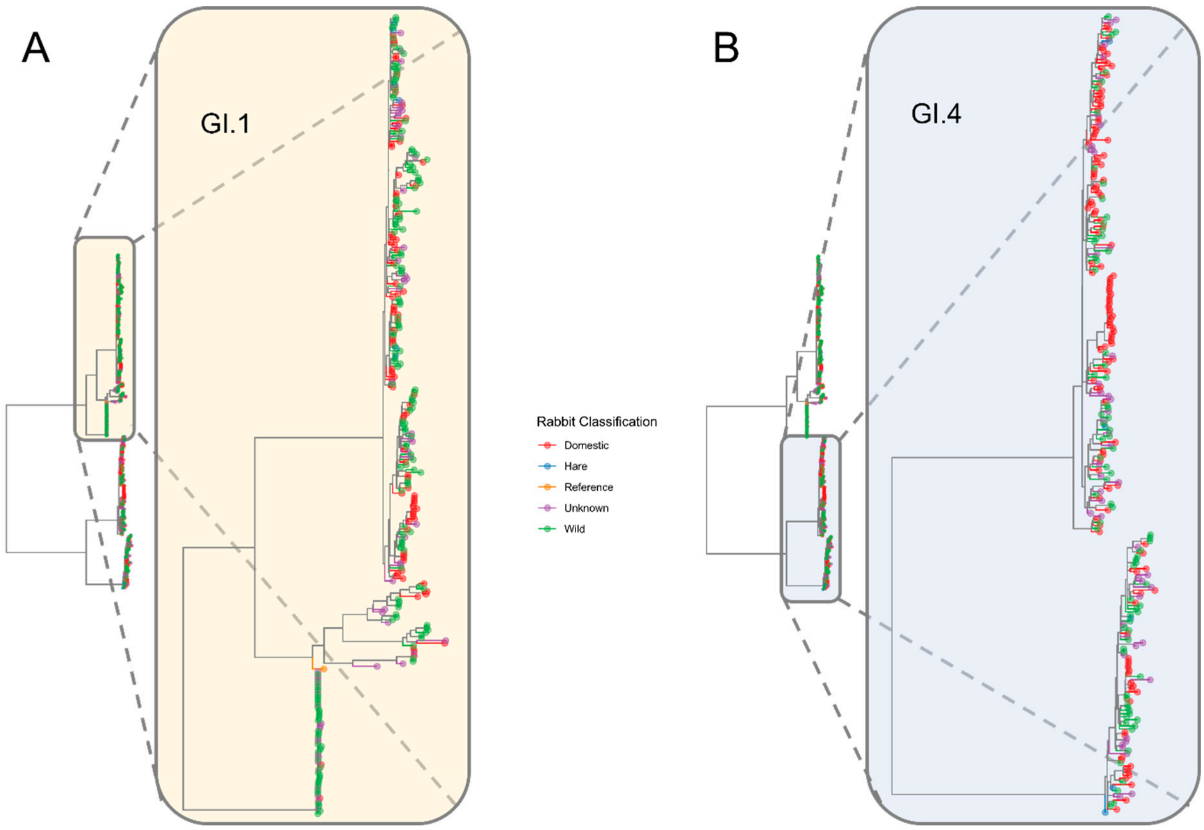

FIG S2. Organisation of RHDV isolates from different sources of leporid samples tested based on non-structural gene genotype. Samples included those obtained from domesticated rabbits (red), hares (blue), unknown (purple) and wild rabbits (green). The reference sequences for GI.1 and GI.4 are coloured in yellow. Respective genotypes are differentiated and highlighted: (A) GI.1 and (B) GI.4.

Table S1: PCR strategy for lagovirus amplification

Fragment	Primer	Sequence (5` to 3`)	Direction	Variant	Position	Reference
**1**	RHDV1	GTGAAARTTATGSCGGCTATGTCGCGC	Fwd	RHDV2	1 – 27	[49]
	RHDV2-1R	TCCCTTGAGTACCTAACGAGCA	Rev	RHDV2	2765 - 2786	This Study
**2**	RHDV2-2L	GGAGTCACTGTCACAACGGT	Fwd	RHDV2	2479 - 2498	This Study
	RHDV2-4c-2R	TTTGCCCTCCATAACATTCACAAA	Rev	RHDV2	5293 - 5316	This Study
**3**	RHDV2-3L	GTTGTCAGGCTAGCCATTGACA	Fwd	RHDV2	4546 - 4567	This Study
	RHDVend	TTTTTTTTTTTTTTTTTTTTTTTTTTTTTTTATA GCTTACTTTAAACTATAAACCCAATTAAACC	Rev	RHDV2	7404 – polyA	[70]
**1**	RHDV1	GTGAAARTTATGSCGGCTATGTCGCGC	Fwd	RHDV2-4c	1 – 27	[49]
	RHDV2-4c-1R	TTGGGGTCGGTGAACACATCA	Rev	RHDV2-4c	2825 - 2855	This Study
**2**	RHDV2-4c-2L	TGGCATGTGATGAGCAACCC	Fwd	RHDV2-4c	2623 - 2642	This Study
	RHDV2-4c-2R	TTTGCCCTCCATAACATTCACAAA	Rev	RHDV2-4c	5293 - 5316	This Study
**3**	RHDV2-4c-3L	CTTGCAGATTGTTGTGAGCAAAC	Fwd	RHDV2-4c	4570 - 4592	This Study
	RHDVend	TTTTTTTTTTTTTTTTTTTTTTTTTTTTTTTATA GCTTACTTTAAACTATAAACCCAATTAAACC	Rev	RHDV2-4c	7404 – polyA	[70]
**1**	RHDV1	GTGAAARTTATGSCGGCTATGTCGCGC	Fwd	RHDV2-4e	1 – 27	[49]
	RHDV2-4e-1R	CTGACGAGTAAACCGCCACATG	Rev	RHDV2-4e	2753 - 2774	This Study
**2**	RHDV2-4e-2L	CAAACCACCCTCATTAACCCCT	Fwd	RHDV2-4e	2524 - 2545	This Study
	RHDV2-4c-2R	TTTGCCCTCCATAACATTCACAAA	Rev	RHDV2-4e	5293 - 5316	This Study
**3**	RHDV2-4e-3L	AGTCACATCCTATGACCATCCTTG	Fwd	RHDV2-4e	4625 - 4648	This Study
	RHDVend	TTTTTTTTTTTTTTTTTTTTTTTTTTTTTTTATA GCTTACTTTAAACTATAAACCCAATTAAACC	Rev	RHDV2-4e	7404 – polyA	[70]
**1**	RHDV1	GTGAAARTTATGSCGGCTATGTCGCGC	Fwd	RHDVa-K5	1 – 27	[49]

	K5_4_R	GCGATCCTGTGAACCAAGTGAG	Rev	RHDVa-K5	1595 - 1616	This Study
2	K5_5_L	TGGTCCTTGGGAAAATCAACATGA	Fwd	RHDVa-K5	1511 - 1534	This Study
	RHDV6	GCCATRGTYGCAAGRTTGACAAGGTGG	Rev	RHDVa-K5	2928 - 2954	[49]
3	RHDV7	GTAYTCAAGRGACCCTGTCCCCGTGG	Fwd	RHDVa-K5	2775 - 2800	[49]
	RHDV10	CATCATCGGRGTCATGGCATACAGGCC	Rev	RHDVa-K5	4828 - 4854	[49]
4	RHDV11	CACCCCATGACYATACTTGACGCCATG	Fwd	RHDVa-K5	4630 - 4656	[49]
	K5_16_R	ATGGTCAATGTCAGCAAACCGG	Rev	RHDVa-K5	6174 - 6195	This Study
5	K5_17_L	CCAAATAGTGGGACTGCAACCA	Fwd	RHDVa-K5	6081 - 6102	This Study
	RHDVend	TTTTTTTTTTTTTTTTTTTTTTTTTTTTTTATAGC TTACTTTAAACTATAAACCCAATTAAACC	Rev	RHDVa-K5	7404 – polyA	[70]

Table S2: PrimalScheme PCR strategy for lagovirus amplification

Fragment	Primer	Sequence (5` to 3`)	Direction	Variant	Position	Reference
1	RHDV1	GTGAAARTTATGSCGGCTATGTCGCGC	Fwd	RHDV2-4e	1 – 27	[49]
	Rec4e_1_R	GGCTCCAAGTTCAGTGAACAGG	Rev		453 - 474	This Study
2	Rec4e_2_L	GGCACTGAACAAAGTCATCCCTT	Fwd		372 - 394	This Study
	Rec4e_2_R	TTGACAACATCGTCGTCGTGTC	Rev		874 - 895	This Study
3	Rec4e_3_L	AAAGTTGTTCATGGCGCATCGA	Fwd		784 - 805	This Study
	Rec4e_3_R	CACCAATCACACCTGCAAAGGT	Rev		1240 - 1261	This Study
4	Rec4e_4_L	GGCCTTCGTTTTCTCCACCATT	Fwd		1155 - 1176	This Study
	Rec4e_4_R	GAGTCAAAGTGGTCAAGGCCAA	Rev		1655 - 1676	This Study
5	Rec4e_5_L	CCGGTGTGGGTAAAACATACTTGG	Fwd		1580 - 1603	This Study
	Rec4e_5_R	AAAAACAAACCCAGGAGCAGGC	Rev		2037 - 2058	This Study
6	Rec4e_6_L	GCCGTTGAAAGTTGGCAAGCTA	Fwd		1933 - 1954	This Study
	Rec4e_6_R	ACACCCTCAACACCGTTCACTA	Rev		2462 - 2483	This Study
7	Rec4e_7_L	CAGTTTGTACTGGGCTGTGTGA	Fwd		2389 - 2410	This Study
	Rec4e_7_R	AGTGTGCTATCTGCCTCCAACT	Rev		2872 - 2893	This Study

**8**	Rec4e_8_L	CCCACCGCGTCTGATAATGTTG	Fwd		2794 - 2815	This Study
	Rec4e_8_R	CGTCAAGTGTCTTGCGTCCTTT	Rev		3283 - 3304	This Study
**9**	Rec4e_9_L	GAGGACCAAGTGACCATTGTCG	Fwd		3208 - 3229	This Study
	Rec4e_9_R	AGCTTGCCTGTGTGTATTGCAA	Rev		3680 - 3701	This Study
**10**	Rec4e_10_L	AGATGTGCTGGCATATGACGGT	Fwd		3597 - 3618	This Study
	Rec4e_10_R	CTTGAAGGGCAGGTTCTCTTGC	Rev		4083 - 4104	This Study
**11**	Rec4e_11_L	TTAGACAAAGTTGATGAGTTCATAGAACG	Fwd		4003 - 4031	This Study
	Rec4e_11_R	ACACAAAAAGTCACCAGCCTTTGA	Rev		4483 - 4506	This Study
**12**	Rec4e_12_L	CTAGGTTTGGGCCAATTGCAGT	Fwd		4409 - 4430	This Study
	Rec4e_12_R	TGGCAGGCAACAAACTAACCAT	Rev		4852 - 4873	This Study
**13**	Rec4e_13_L	GGCCTTCACTGTTCAAACCTGT	Fwd		4771 - 4792	This Study
	Rec4e_13_R	CGACACGTTTACGATCTGCCAA	Rev		5248 - 5269	This Study
**14**	Rec4e_14_L	ATCAAGCCAGAACTAGAACGTCAA	Fwd		5173 - 5196	This Study
	Rec4e_14_R	ACCCAGCGACTATAAACCGGAA	Rev		5659 - 5680	This Study
**1**	RHDV1	GTGAAARTTATGSCGGCTATGTCGCGC	Fwd		1 – 27	[49]
	Rec4c_1_R	CATCAGCTTCCTGCCTTCATCC	Rev		510 -531	This Study
**2**	Rec4c_2_L	TTTGAAGGCGAAGTTGACGACC	Fwd		433 -454	This Study
	Rec4c_2_R	GCAAGAAGGTTGACGGGTTTGA	Rev		899 -920	This Study
**3**	Rec4c_3_L	TTGACCCTCTGAAAACCCTTGC	Fwd		812 -833	This Study
	Rec4c_3_R	AAGTCTTTTGCCACCATGTGCT	Rev		1292 -1313	This Study
**4**	Rec4c_4_L	GAAAGGGGCTGGAAAACTCACC	Fwd	RHDV2-4c	1218 -1239	This Study
	Rec4c_4_R	CCCGTGTACGAGTCAAAATGGT	Rev		1664 -1685	This Study
**5**	Rec4c_5_L	GGCGTTGGCAAAACATACTTGG	Fwd		1582 -1603	This Study
	Rec4c_5_R	CCTGGGTGCAACTTGTGTCTTT	Rev		2076 -2097	This Study
**6**	Rec4c_6_L	CATGTCCCATCTCACTTTCTTGGT	Fwd		1998 -2021	This Study
	Rec4c_6_R	GCAAGATGTTGTCCACGGTTGT	Rev		2491 -2512	This Study

**7**	Rec4c_7_L	GTGAACATGACAAACCAACTCGG	Fwd		2407 -2429	This Study
	Rec4c_7_R	ACAAACAGGTGTGCAATCTGCC	Rev		2879 -2900	This Study
**8**	Rec4c_8_L	TATCTGACAACGTAGACCGGGG	Fwd		2801 -2822	This Study
	Rec4c_8_R	TCAAGTGTCTTGCGTCCCTTTG	Rev		3281 -3302	This Study
**9**	Rec4c_9_L	TTGAGACCTGACGAGGACCAAG	Fwd		3196 -3217	This Study
	Rec4c_9_R	ACTCATCGTACAACGGCAAACC	Rev		3646 -3667	This Study
**10**	Rec4c_10_L	AGAAGACATTGTCAGACTCAACCAA	Fwd		3569 -3593	This Study
	Rec4c_10_R	TGCAGCACGCTCGATAAATTCA	Rev		4017 -4038	This Study
**11**	Rec4c_11_L	ACTGCACGCTGATGAACATAGC	Fwd		3944 -3965	This Study
	Rec4c_11_R	ACTGCAATTGGTCCAAACCTGG	Rev		4409 -4430	This Study
**12**	Rec4c_12_L	GTTACTCTGGGGTTGTGACGTG	Fwd		4326 -4347	This Study
	Rec4c_12_R	ACTCCATCATCACCGTACGTGT	Rev		4811 -4832	This Study
**13**	Rec4c_13_L	GGTTACTTTGGTCAGCAGCAGT	Fwd		4727 -4748	This Study
	Rec4c_13_R	AACTTCGTGTAAGCCTGCTGAC	Rev		5192 -5213	This Study
**14**	Rec4c_14_L	ACGGCACATGACACTTGAAGAG	Fwd		5061 -5082	This Study
	Rec4c_14_R	GCGTCTGCAACTGACCATGTAA	Rev		5537 -5558	This Study
**15**	RHDV2_15_L	CCTGGTGTTGTGGCCACTACTA	Fwd	RHDV2, RHDV2-4e, RHDV2-4c	5398 - 5419	This Study
	RHDV2_15_R	TTGTAAACGCTCAGGACCAACG	Rev		5870 - 5891	This Study
**16**	RHDV2_16_L	CGAACCAGTCACCATCACCATG	Fwd		5793 - 5814	This Study
	RHDV2_16_R	CTGGAGCAATTTGGGAGATGGG	Rev		6283 - 6304	[19]
**17**	RHDV2_17_L	GCAAGTTTCCCTGGAAGCAGTT	Fwd		6208 - 6229	This Study
	RHDV2_17_R	GCGAACATGATGGGTGTGTTCT	Rev		6674 - 6695	This Study
**18**	RHDV2_18_L	CACCCCAAACAGTAGTGCCATT	Fwd		6585 - 6606	This Study
	RHDV2_18_R	CCTGCAAGTCCAAGTCCAACAA	Rev		7047 - 7068	This Study
**19**	RHDV2_19_L	AACCACCCTCATTGATCTGTCAGA	Fwd		6927 - 6950	This Study

	RHDVend	TTTTTTTTTTTTTTTTTTTTTTTTTTTTATAG CTTACTTTAAACTATAAACCCAATTAAACC	Rev		7404 – polyA	[70]

19. Mahar, J.E.; Jenckel, M.; Huang, N.; Smertina, E.; Holmes, E.C.; Strive, T.; Hall, R.N. Frequent intergenotypic recombi-nation between the non-structural and structural genes is a major driver of epidemiological fitness in caliciviruses. Virus Evol. 2021, 7, veab080. https://doi.org/10.1093/ve/veab080.
44. Statistics, A.B.o. National, state and territory population. . Available online: https://www.abs.gov.au/statistics/people/population/national-state-and-territory-population/sep-2022 (accessed on September 30).
49. Elsworth, P.; Cooke, B.D.; Kovaliski, J.; Sinclair, R.; Holmes, E.C.; Strive, T. Increased virulence of rabbit haemorrhagic disease virus associated with genetic resistance in wild Australian rabbits (Oryctolagus cuniculus). *Virology* **2014**, *464-465*, 415-423, doi:10.1016/j.virol.2014.06.037.
70. Hall, R.N.; Capucci, L.; Matthaei, M.; Esposito, S.; Kerr, P.J.; Frese, M.; Strive, T. An in vivo system for directed experimental evolution of rabbit haemorrhagic disease virus. *PLoS One* **2017**, *12*, e0173727-e0173727, doi:10.1371/journal.pone.0173727.
